# cuRRBS: simple and robust evaluation of enzyme combinations for reduced representation approaches

**DOI:** 10.1093/nar/gkx814

**Published:** 2017-09-19

**Authors:** Daniel E. Martin-Herranz, António J. M. Ribeiro, Felix Krueger, Janet M. Thornton, Wolf Reik, Thomas M. Stubbs

**Affiliations:** 1 European Molecular Biology Laboratory, European Bioinformatics Institute, Wellcome Genome Campus, Hinxton CB10 1SD, UK; 2 Bioinformatics Group, The Babraham Institute, Cambridge CB22 3AT, UK; 3 Epigenetics Programme, The Babraham Institute, Cambridge CB22 3AT, UK; 4 Centre for Trophoblast Research, University of Cambridge, Cambridge CB2 3EG, UK; 5 Wellcome Trust Sanger Institute, Hinxton CB10 1SA, UK

## Abstract

DNA methylation is an important epigenetic modification in many species that is critical for development, and implicated in ageing and many complex diseases, such as cancer. Many cost-effective genome-wide analyses of DNA modifications rely on restriction enzymes capable of digesting genomic DNA at defined sequence motifs. There are hundreds of restriction enzyme families but few are used to date, because no tool is available for the systematic evaluation of restriction enzyme combinations that can enrich for certain sites of interest in a genome. Herein, we present customised Reduced Representation Bisulfite Sequencing (cuRRBS), a novel and easy-to-use computational method that solves this problem. By computing the optimal enzymatic digestions and size selection steps required, cuRRBS generalises the traditional MspI-based Reduced Representation Bisulfite Sequencing (RRBS) protocol to all restriction enzyme combinations. In addition, cuRRBS estimates the fold-reduction in sequencing costs and provides a robustness value for the personalised RRBS protocol, allowing users to tailor the protocol to their experimental needs. Moreover, we show *in silico* that cuRRBS-defined restriction enzymes consistently out-perform MspI digestion in many biological systems, considering both CpG and CHG contexts. Finally, we have validated the accuracy of cuRRBS predictions for single and double enzyme digestions using two independent experimental datasets.

## BACKGROUND

With the advent of next-generation sequencing (NGS) scientists are studying the biology of life at unprecedented resolution (1). Unfortunately, owing to the large size of many commonly studied genomes (human, mouse and tobacco plant for example are all >2.5 Gbp in size) (2–4), it is often still prohibitively expensive to conduct whole genome sequencing at high coverage. This creates a trade-off that negatively impacts the number of replicates that can be included and, therefore, it challenges the statistical power and the reproducibility of the studies (5,6). This is true in particular for DNA methylation, where differentially methylated regions (DMRs) are typically called by identifying changes as small as 10% and where 70–80% of the reads of Whole Genome Bisulfite Sequencing (WGBS) methods contain little to no relevant information on the DNA methylation status (7).

To address these cost inefficiencies, many methods have been developed to reduce the number of fragments that need to be sequenced for a given biological system or WGBS validation experiment (8–25). These methods can be broadly split into those that positively select for genomic fragments of interest and those that deplete for fragments that are not of interest. Positive selection-based methods involve the sites of interest being enriched from the background. This usually occurs through pull-down of these sites via an antibody (e.g. anti-5mC antibody), a recombinant binding protein (e.g. methyl-CpG-binding domains (MBD)), RNA baits for the sites of interest, array-based approaches (e.g. EPIC array in human) or PCR-based approaches (26). These methods have many limitations, including enrichment biases, complex protocols and difficulties in quantification (27).

Current evidence shows that depletion-based methods do not have enrichment biases, tend to be simpler and are more readily quantifiable (26,27). The most common depletion-based approaches use restriction enzymes to exploit the fact that the nucleotide composition in a given genome is non-random (28) and that the fragment lengths produced from a given digestion will thus reflect this. In the case of 5-methylcytosine (5mC), the most common depletion-based method is Reduced Representation Bisulfite Sequencing (RRBS) using the methylation-insensitive restriction enzyme MspI (with the recognition sequence C|CGG), although enzymes such as BglII, XmaI and Taq^α^I have also been used (29–31). RRBS has proven extremely useful for cost-effective, global studies of DNA methylation (10,32,33), capturing around 10% of CpG sites within mammalian genomes but with up to a 30-fold reduction in the number of fragments sequenced in comparison to WGBS (34).

Whilst restriction enzyme-based approaches are versatile, simple and cost-effective, the utility of the MspI-based RRBS approach is limited to a specific subset of CpG sites in the genome, mainly found within CpG islands and promoters (32). To allow researchers to optimise the protocol used for their specific experiment, we have developed a new computational method called customised Reduced Representation Bisulfite Sequencing (cuRRBS). cuRRBS generalises the problem of genomic enrichment with restriction enzymes by allowing the user to define both the genome and the particular sites of interest, before outputting the optimal enzyme combinations and size ranges to target these sites. In addition, cuRRBS provides the user with a variety of metrics to compare the various suggested protocols, including an estimate of the fold-reduction in sequencing costs compared to WGBS and a robustness value to assess the impact of experimental error in the size selection step.

Here, we have tested the enrichment ability of cuRRBS in several biological systems, with sites in both CpG and CHG contexts and multiple species, to showcase the generalisability and utility of the software (35–41). In addition, we take advantage of two recently published independent RRBS datasets to demonstrate the accuracy of the software predictions in both single and double enzyme experimental settings (30,31).

We hope that cuRRBS will be useful both as a tool for designing cost-effective, genome-wide studies in the future but also for validating previous results from whole genome approaches in a simple, cheap and timely fashion, something that at present is not possible.

## MATERIALS AND METHODS

### Restriction enzymes annotation

All the information regarding the commercially-available restriction enzymes that are used by cuRRBS was extracted from REBASE (42,43). Restriction enzymes were grouped in isoschizomer families (i.e. enzymes that recognise the same sequence and generate identical fragment length distributions) and each enzyme was manually annotated for different types of methylation-sensitivity (CpG, CHG, CHH). Only isoschizomer families that contained at least one methylation-insensitive enzyme were considered for the examples described in this manuscript.

### Genome assemblies and genomic annotation

All the analyses presented here were performed in the following genome assemblies: *Homo sapiens* (hg38), *Mus musculus* (mm10) and *Arabidopsis thaliana* (TAIR10). Scaffolds not assembled into the main chromosomes were discarded. Genomic annotation for the human genome (hg38) was obtained from GENCODE (v25, basic gene annotation) (44), with the exception of *CpG islands* (CGIs), which were extracted from the UCSC Genome Browser (45). *GC content* and *CpG content* were calculated, around each restriction enzyme cleavage site, taking windows of ±25 bp and ±500 bp respectively. For each enzyme, the mean of all cleavage sites was calculated to obtain the *mean GC content* and the *mean CpG content. Intragenic* regions were defined as those regions within ±2.5 kb of a protein-coding gene, whilst the rest of the genome was considered to be *intergenic. CpG shores* were defined as regions 0 to 2 kb away from CGIs in both directions and *CpG shelves* as regions 2 to 4 kb away from CGIs in both directions (46). *Promoters* were defined as encompassing a 3 kb region (2.5 kb upstream and 0.5 kb downstream of the TSS) relative to the TSS of all protein-coding transcripts in GENCODE, similar to the strategy used in Taher *et al.* (47). Genomic annotation for the CGIs in the mouse genome (mm10) was also obtained from the UCSC Genome Browser (45). All annotations were handled using the *pybedtools* library (48,49).

### Performing *in silico* digestions of a given genome

We used the *Restriction* package from Biopython v1.68 to digest the different genomes with the appropriate restriction enzymes *in silico* (50). Only the first member of a given isoschizomer family (which contained at least one methylation-insensitive enzyme) was processed to avoid redundant computations. The output of the *in silico* digestions was stored (pre-computed files) and subsequently read by cuRRBS when needed to reduce the computational time (see ‘cuRRBS heuristics and computational efficiency’). When assessing enzyme combinations, the information from the appropriate individual pre-computed files (i.e. the genomic coordinates where the enzyme theoretically cuts) were combined by the software to compute all the necessary variables.

### cuRRBS’ enzyme flexibility

To ensure the user has full control over the enzymes that cuRRBS will use to derive the desired enrichments, one of the inputs given to cuRRBS is an enzyme annotation file. This file contains the desired isoschizomer families that the user wishes to be tested by cuRRBS. In the GitHub repository we have already defined enzyme annotation files for enzymes that are methylation-insensitive in a CG context and in CG, CHG and CHH contexts (51). However, it is also possible for the user to define a personalised set of enzymes by providing a self-generated annotation file. This can be useful, for instance, to reduce the chance of any star activity in the reported cuRRBS protocols.

In addition, the output file from cuRRBS contains, by default, 30 cuRRBS protocols that would enrich for the user's sites of interest. Therefore, the user can determine which enzyme combination and size range would be the simplest and most appropriate for the given application. This provides the user with the opportunity to consider experimental factors that may complicate the protocol, such as buffer compatibility and whether consecutive digestions would be required.

### Calculating cuRRBS main variables

cuRRBS makes use of several variables in order to find the best enzyme combination and size range which enriches for a certain set of genomic sites defined by the user (see ‘cuRRBS overview’). For a given enzyme (combination) and size range, the *Enrichment Value* (*EV*) can be calculated as:

\begin{equation*}EV = - {\log _{10}}\left( {\frac{{Score}}{{NF}} \cdot \frac{n}{{\max \_Score}}} \right)\end{equation*}

Here, *NF* is the number of genomic fragments that will theoretically be sequenced (i.e. those whose lengths after the *in silico* digestion are within the size range); *n* is the total number of sites of interest; *max_Score* is the *Score* obtained if all the sites of interest were sequenced and:

\begin{equation*}Score = \sum\limits_{i = 1}^n {{w_i} \cdot {\gamma _i}} \end{equation*}

where *w_i_* is the weight of the *i*th site of interest and *γ_i_* is 1 if the *i*th site would be theoretically sequenced (i.e. present in a size selected fragment and ≤ *read length* base pairs away from one of the ends of the fragment) and 0 otherwise. Since the objective of cuRRBS is to maximize the *Score* while minimizing the *NF*, the best results will be obtained when *EV* is minimized.

cuRRBS output also contains other variables that may be of interest to the user (see ‘cuRRBS overview’). The *Cost Reduction Factor* (*CRF*) for a given cuRRBS protocol can be calculated as:

\begin{equation*}CRF = \frac{{N{F_{ref}}}}{{NF}} = \frac{{g/r}}{{NF}}\end{equation*}

where *NF_ref_* is the estimated number of fragments that would be sequenced in a Whole Genome Bisulfite Sequencing (WGBS) experiment, that can be roughly calculated as the genome size (*g*) divided by the read length (*r*).

Furthermore, the *robustness* (*R*) of a given enzyme (combination) is calculated as:

\begin{equation*}R = {e^{ - \theta }}\end{equation*}

with

\begin{equation*}\theta = \frac{{\mathop \sum \nolimits_{x \in \left\{ {a - \delta ,a,a + \delta } \right\}} \mathop \sum \nolimits_{y \in \left\{ {b - \delta ,b,b + \delta } \right\}} \left| {E{V_{x,y}} - E{V_{a,b}}} \right|}}{{E{V_{a,b}}}}\end{equation*}

where *EV_a,b_* is the *EV* for the optimal size range (*a*: lower limit in size range, *b*: breadth) and *δ* is the experimental error (in bp) that is assumed during the size selection step. The *robustness* will take values in the interval (0,1], with higher values identifying robust cuRRBS protocols.

### Flexible user-defined cuRRBS parameters

cuRRBS contains a number of user-defined parameters to ensure the greatest possible flexibility and ease of use. A table of these parameters is provided to highlight the versatility that the user has and why such versatility is useful (Table 1).

**Table 1.** Flexible user-defined cuRRBS parameters

cuRRBS parameter (abbrev.)	Significance	Default	Range
Enzymes to check (-e)	Defines the enzymes (isoschizomer families) that cuRRBS will look at	-	-
Annotation for the sites of interest (-a)	Allows identification and weighting of the sites of interest	-	-
Read length (-r)	Defines the positions in the theoretical fragments that can be ‘seen’ after sequencing	-	30–300
Adapters size (-s)	Ensures correct experimental size selection	-	-
C_Score constant (-c)	Sets the minimum acceptable *Score*	-	0–1
Genome size (-g)	Needed to calculate the *CRF*	-	-
C_NF/1000 constant (-k)	Sets the minimum acceptable *CRF*	0.2	0–1
Experimental error (-d)	Sets the assumed experimental error (*δ*)	20	5–500
Size range breadth (-b)	Constrains the breadth of the size range	980	-
Output size (-t)	Defines the number of cuRRBS protocols the user can compare	30	-
Site IDs (-i)	Enables the identification of the recovered sites of interest	No	-

This table details the flexible user-defined parameters that cuRRBS will accept as arguments. The cuRRBS parameter full name and command-line abbreviation (in brackets) are provided alongside a simplified description of the significance of these arguments to the user. Where applicable, the defaults and ranges are also detailed.

### cuRRBS heuristics and computational efficiency

cuRRBS employs several strategies to reduce the computational time needed in each run:

1. Restriction enzymes are grouped in isoschizomer families. Since isoschizomers generate the same genomic digestions, only one member of each family needs to be processed.
2. *In silico* digestions are read from pre-computed files. Digesting the genomes would be a limiting factor in the cuRRBS pipeline. The user can download the pre-computed files (51) and the information that they contain is read every time that an enzyme needs to be assessed.
3. The number of size ranges that are sampled is minimised. Since the experimental size selection step is generally imperfect, size ranges are sampled with a sliding window whose ‘resolution’ is equivalent to the experimental error specified by the user.
4. Parallelization. cuRRBS can use several cores to decrease the CPU time.

Moreover, we have observed that, in many enzyme combinations, one of the enzymes is providing most of the enrichment for the sites of interest, while the second one complements the targeting. Therefore, it would be possible to implement a ‘heuristic’ mode, where only those enzymes that perform well individually are used as ‘seeds’ to construct combinations (as opposed to the current implementation, where all the enzyme combinations are checked exhaustively). This could further reduce the computational time, especially if combinations of more than two enzymes were being evaluated.

The CPU time required by cuRRBS depends on several parameters, including the number of enzymes checked, the experimental error, the number of sites of interest or the genome size (Supplementary Figure S1A–D). The RAM used will be approximately equal to the size of the pre-computed files that are read by the software. A standard cuRRBS run (e.g. for a few thousand sites of interest in the human genome, checking 128 CpG methylation-insensitive isoschizomer families) takes ∼0.5–1 h and uses around 4GB RAM, which allows the user to easily run it on a dual-core laptop or desktop computer.

### Obtaining the sites of interest for different biological systems

We have tested *in silico* the ability of cuRRBS to enrich for the sites of interest in a selection of different biological systems where DNA methylation has an important functional role. In some of these systems, described below, previous analysis was performed in order to obtain the genomic coordinates for the sites:

1. Exon-intron boundaries in human. Exons and introns were obtained from protein-coding genes using GENCODE annotation data. Those CpG sites that were found within ± 5 bp of a canonical splice site (5′-GT, 3′-AG) were selected.
2. Epigenetic clock in human. These sites were obtained from Horvath (37). Briefly, these sites make up an elastic-net regression model that is able to predict chronological and biological age in humans. These sites were lifted over to hg38 for the analysis conducted in this paper (52).
3. Canonical and placental imprints in human. These loci were obtained from Hanna *et al.*, 2016 (35). These sites were lifted over to hg38 and the CpG sites were then extracted for the analysis conducted in this paper (52).
4. CTCF binding sites in human. We obtained the CpG sites that overlap with *in vivo* CTCF binding sites. Peaks from sites that seem to be affected by methylation (upregulated, reactivated) were kindly provided by Maurano (39). We scanned the peaks for high-scoring motifs according to the CTCF JASPAR model (53). Finally, we extracted those CpGs that were found in positions 5 and 15 of the motif, whose methylation status is supposed to influence the binding of the transcription factor (39).
5. Induced pluripotent stem cell (iPSC) demethylated and maintained sites in mouse. These were obtained as described previously (36), with an additional filter for magnitude of methylation change. In brief, a background model was obtained by calculating the global mean for the ending methylation value for each starting methylation value, comparing mouse embryonic fibroblasts (MEFs) to iPSCs. Afterwards, a binomial test was used on individual probes for their ending methylation measured against the mean for their starting methylation, to obtain p-value < 0.05 differentially methylated regions (DMRs). Adjusted p-values were calculated using a pairwise *t*-test with a Benjamini–Hochberg correction and only probes accounting for more than 50% loss of methylation relative to the genome average loss (from MEFs to iPSCs, commonly located at super-enhancer regions) or more than 50% gain in methylation relative to the genome average loss (from MEFs to iPSCs, commonly located at intracisternal A-particles) were used in this analysis.
6. NRF1 binding sites in mouse. We obtained the CpG sites that overlap with *in vivo* NRF1 binding sites in mouse. ChIP-seq data was processed as described in the original publication (41), where peaks were called using Peakzilla (54). We took as our final set of peaks the overlap between the two TKO replicates. Next, we scanned the peaks for high-scoring motifs according to the NRF1 JASPAR model (53). Finally, we extracted those CpGs that were found in positions 2 and 8 of the motif, whose methylation status is supposed to influence the binding of the transcription factor (41).
7. CHG sites in *Arabidopsis thaliana*. Non-CpG DMRs arising from the epigenomic diversity between *Arabidopsis thaliana* accessions were obtained from Kawakatsu *et al.* (38). The coordinates for C sites in non-CpG context were extracted.

In all the cases the sites were equally weighted (*w_i_* = 1), with the exception of the human epigenetic clock system, where the sites were assigned the absolute value of the weights in the linear model (37).

All the site annotation files are provided as Supplementary Tables S1–S9.

### Running cuRRBS for different *in silico* systems

cuRRBS was run in the different systems described above using the default parameters (*k* = 0.2, *d* = 20, *b* = 980, *t* = 30), for a *read length* (*r*) of 75 bp and a *Score threshold* (*c*) of 0.25. In the mouse and human examples we considered 128 isoschizomer families that contained enzymes that were not sensitive to CpG methylation. In the case of *Arabidopsis thaliana* we used 28 isoschizomer families that contained enzymes that were not sensitive to 5mC in any context (CG, CHG, CHH).

### Mapping of RRBS samples

XmaI-RRBS data generated on the Ion Torrent platform (30) and MspI&Taq^α^I-RRBS data generated on the Illumina HiSeq platform (31) were quality-trimmed using Trim Galore (www.bioinformatics.babraham.ac.uk/projects/trim_galore/) and had base pairs removed from the 3′ end to avoid including filled-in nucleotides with artificial methylation states (the filled-in XmaI, MspI and Taq^α^I cut sites include the nucleotide sequence CCGG, CG and CG respectively). The data was then mapped to the human genome (for XmaI data, parameters: –non_directional) or the mouse genome (for MspI&Taq^α^I data, parameters: –directional) using Bismark (0.18.0) (55). In each of the two cases data from different experiments or replicates was merged into the same FASTQ file prior to quality trimming.

### Estimating cuRRBS’ sensitivity and specificity

We assessed the performance of cuRRBS predictions in two independent experimental datasets (30,31) (see ‘Experimental validation of cuRRBS’ in Results and discussion). We ran cuRRBS fixing the theoretical size ranges tested to the ones reported in the publications (30,31) and we used as our sites of interest the CpGs that overlapped with CpG islands (CGI-CpGs) in the human (30) and the mouse genomes (31) respectively. From the cuRRBS output files, we recovered the IDs of the sites that should be theoretically sequenced. Moreover, using the experimental RRBS data (30,31), we could obtain the IDs of the sites that were actually sequenced (filtered by a given depth of coverage threshold). Afterwards, we calculated the following variables for each one of the datasets:

1. True positives (*TP*): number of CGI-CpGs that cuRRBS predicted to be sequenced and were indeed found in the RRBS data.
2. True negatives (*TN*): number of CGI-CpGs that cuRRBS predicted to be absent and were not found in the RRBS data.
3. False positives (*FP*): number of CGI-CpGs that cuRRBS predicted to be sequenced but were not found in the RRBS data.
4. False negatives (*FN*): number of CGI-CpGs that cuRRBS predicted to be absent but were found in the RRBS data.

Finally, we estimated the sensitivity and specificity, for a given dataset, as follows:

\begin{equation*}Sensitivity = \frac{{TP}}{{TP + FN}} \cdot 100\end{equation*}

\begin{equation*}Specificity\ = \frac{{TN}}{{FP + TN}}\ \cdot 100\end{equation*}

## RESULTS AND DISCUSSION

### Restriction enzyme digestion as a tool for genomic enrichment

**Figure 1.**
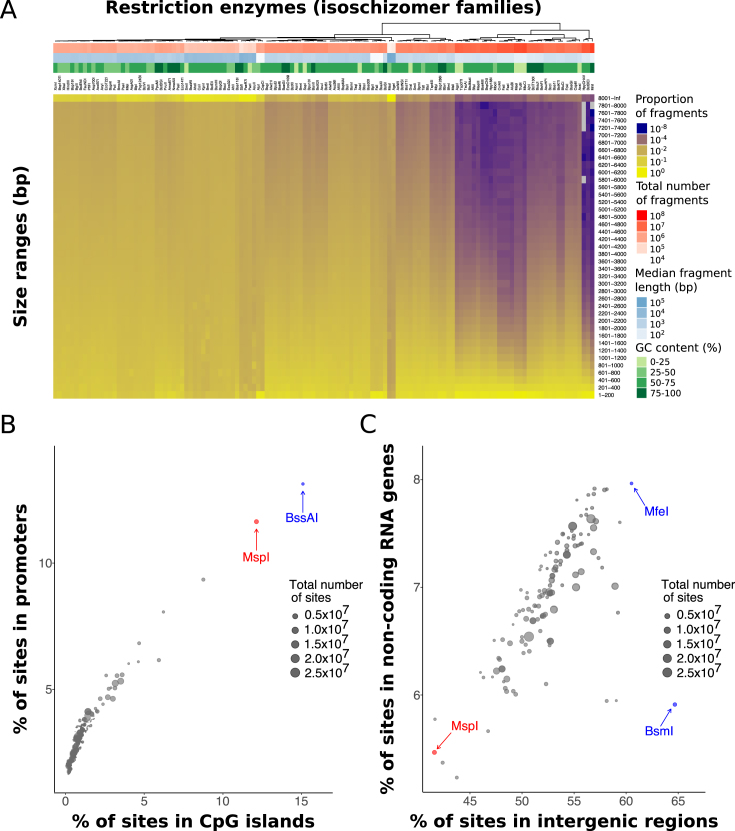
Restriction enzyme digestion as a tool for genomic enrichment. (**A**) Heatmap showing the fragment length distributions generated by different restriction enzymes in the human genome (hg38). Each column represents the distribution for an isoschizomer family of restriction enzymes that contains at least one member which is methylation-insensitive in a CpG context. The distributions are binned in *size ranges* of 200 bp, ordered as they would appear in an electrophoretic gel. Additional row annotations on top of the heatmap contain information regarding the total number of fragments (in red) and the median fragment length (in blue) produced by each *in silico* digestion, together with the GC content of the recognition motif in the isoschizomer family (in green). Legend is displayed on the right hand side. (**B**) Scatterplot showing the percentage of cleavage sites from different restriction enzymes that overlaps with CpG islands (X-axis) and promoters (Y-axis) in the human genome (hg38). The size of the circles represents the total number of cleavage sites generated by each enzyme. The enzymes MspI and BssAI are highlighted in red and blue respectively. Legend is displayed on the right hand side. (**C**) Scatterplot showing the percentage of cleavage sites from different restriction enzymes that overlap with intergenic regions (X-axis) and non-coding RNA genes (Y-axis) in the human genome (hg38). The size of the circles represent the total number of cleavage sites generated by each enzyme. The enzyme MspI is highlighted in red. The enzymes BsmI and MfeI are both highlighted in blue. Legend is displayed on the right hand side.

Restriction enzymes represent an incredibly effective tool for the enrichment of certain sites of interest in a genome. This is possible due to the wide variety of motifs that commercially-available restriction enzymes can recognise (Supplementary Figure S2A and B) combined with the non-random nature of the genome composition itself. Supplementary Figure S2A and B highlight that this motif diversity is driven both by the sequence composition (GC content) and the length of the recognition sequence. Thus, different restriction enzymes will generate different fragment length distributions, dependent upon how frequently their recognition site is present in a given genome (Figure 1A, Supplementary Figure S2C).

In DNA methylation studies the most common application is the use of MspI (cutting at C|CGG) in RRBS (Reduced Representation Bisulfite Sequencing), which is used to enrich for CG dinucleotides (CpGs) contained in promoters and CpG islands (32) (Figure 1B). However, in many cases, MspI is by no means the most effective restriction enzyme that could be used. For instance, MspI would be a poor restriction enzyme to choose for the enrichment of CpGs found in intergenic regions or non-coding RNA genes, which would be far better enriched for using BsmI or MfeI respectively (Figure 1C). In fact, it turns out that across many genomic features MspI is rarely the most optimal methylation-insensitive restriction enzyme (Supplementary Figure S2D).

Previous studies have tested the potential of other restriction enzymes and enzyme combinations to expand the range of CpG sites that can be targeted in a genome (8–11,28,30,56,57). However, to our knowledge, there is currently no computational method that systematically explores the capacity of all commercially-available restriction enzymes to generate ‘personalised’ reduced-representations of the genome whilst minimising the experimental cost (Supplementary Figure S2E).

### cuRRBS overview

**Figure 2.**
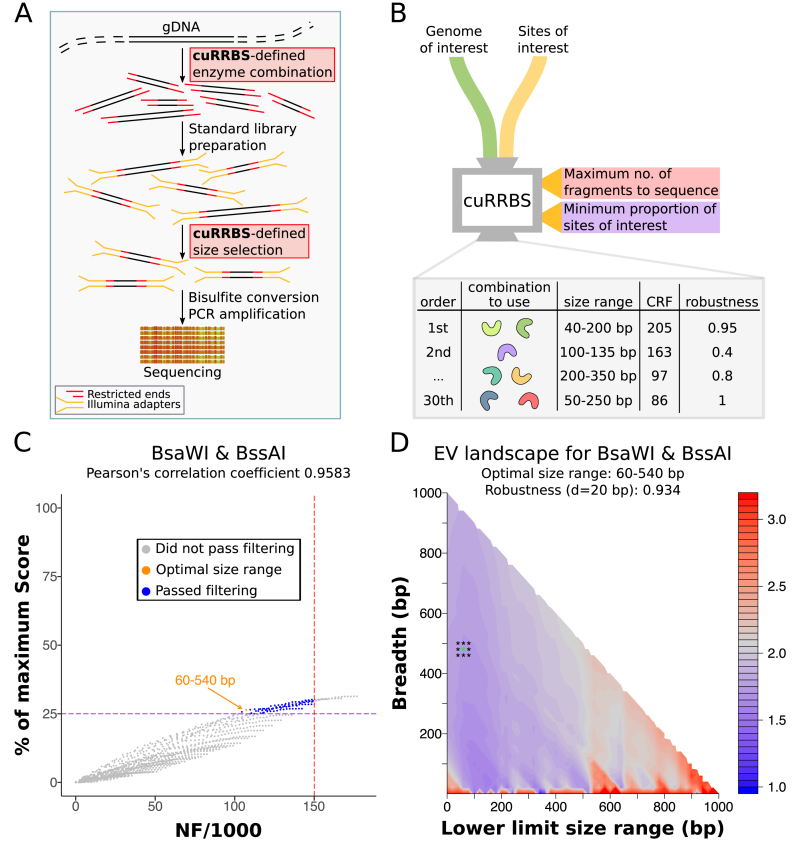
cuRRBS overview. (**A**) Outline of an RRBS protocol. Highlighted are the two steps that would be modified according to the output produced by cuRRBS (i.e. the restriction enzymes used for the genomic digestion and the size selection). Legend is displayed on the bottom left. (**B**) Schematic of cuRRBS. Highlighted are the two main inputs required for the software and the two *thresholds* that the user has to define (red and purple tags). The default output for cuRRBS is a table containing the top hits (restriction enzyme combination and size range) along with additional information that might be useful to the user (such as *Cost Reduction Factor* and *robustness*). (**C**) Scatterplot showing the trade-off between the number of fragments (*NF*) and the *Score* for the best enzyme combination (BsaWI & BssAI) that targets the CpGs present in the human placental-specific imprinted regions (35). *NF* is divided by 1000 for visualization purposes. Each point represents a different *size range*. Shown in dark blue and grey are the size ranges that would and would not pass filtering respectively. Shown in orange is the optimal size range in the filtered search space. The dotted lines depict the *thresholds* that need to be specified by the user (red: maximum *NF*; purple: minimum percentage of the maximum *Score*). In this mock example we specified an *NF threshold* of 150 000 fragments and a *Score threshold* of 25% of the maximum *Score*. Legend is displayed below the plot title. (**D**) Contour plot that depicts how the *robustness* (*R*) variable is calculated for the optimal enzyme combination (BsaWI & BssAI; size range: 60–540 bp) that targets the CpGs present in the human placental-specific imprinted regions (35). *Enrichment values* (*EV*s) are calculated for all possible size ranges in order to create an ‘*EV* landscape’. In this landscape, cuRRBS finds the size range with the lowest *EV* that still satisfies the *thresholds* (asterisk in green). Afterwards, cuRRBS samples *EV*s around the optimum (asterisks in black). The points that are sampled depend on the experimental error (in this case, *δ =* 20 bp). A high *robustness* value means that the sampled *EV*s do not change a lot when compared to the optimum, which implies that cuRRBS prediction will not be greatly affected by experimental errors during the size selection step.

We have developed a novel computational method (cuRRBS) that determines the optimal combination of restriction enzymes and size range to enrich for any given set of sites of interest in any genome. In other words, by modifying two of the steps in the original RRBS protocol (Figure 2A), cuRRBS generalises RRBS.

The software takes as input the genomic coordinates that the user wants to target (Figure 2B, Supplementary Figure S3A). Afterwards, cuRRBS assesses *in silico* the potential of all single enzymes and double-enzyme combinations to enrich for the sites of interest using the following two key variables (see Materials and Methods):

1. *NF*, which reflects the theoretical number of fragments that will be sequenced after the size selection step. Assuming that the sequencing cost is proportional to *NF*, cuRRBS attempts to minimise this value.
2. *Score*, which reflects the theoretical number of sites of interest that will be sequenced after the size selection step. cuRRBS attempts to maximise this value.

The *NF* and *Score* variables are positively correlated with one another, such that the more genomic fragments sequenced, the more sites of interest are likely to be contained within the reduced representation (Figure 2C, Supplementary Figure S3B). However, this relationship disappears at higher *NF* values, where the *Score* variable becomes saturated such that any additional fragments sequenced will result in a reduction in the overall enrichment of the sites of interest. This *Score* saturation at high *NF* is mainly due to additional sites of interest being buried within long fragments that will not be sequenced due to limitations in the read length (cuRRBS parameter –*r*, see Table 1). For a given enzyme or enzyme combination, the *NF* and the *Score* variables depend on the *size range* chosen, since only the genomic fragments within the size range will be present in the reduced representation of the genome.

cuRRBS requires that the user sets *thresholds* for the maximum *NF* (i.e. minimum *CRF*) and minimum *Score* that would be acceptable for a given application (Figure 2B, Supplementary Figure S3A). These *thresholds* allow cuRRBS to search through all possible *size ranges* for a given enzyme or enzyme combination and to find the one that minimises the *Enrichment Value* (*EV*), a variable that combines both *NF* and *Score* into a single number (see Materials and Methods). cuRRBS repeats this procedure for every single enzyme and enzyme combination and reports those with the best hits (i.e. those with the lowest *EV*s) (Supplementary Figure S3A).

The output file contains the best scoring enzymes with their correspondent size ranges and some other useful variables for each one of the hits, such as (see Materials and Methods):

1. *Cost Reduction Factor* (*CRF*). This estimates the theoretical fold-reduction in sequencing costs for the cuRRBS protocol when compared to Whole Genome Bisulfite Sequencing (WGBS).
2. *Robustness* (*R*). This assesses how much the cuRRBS prediction varies if a slightly different size range is used (Figure 2D). The results for robust enzymes will not be greatly affected as a consequence of experimental error during the size selection step. This will help the user to make an informed decision on which enzyme combination to choose for the system of interest (Supplementary Figure S3C).

### Running cuRRBS in different biological systems

cuRRBS provides a way to effectively interrogate DNA methylation in any biological system for which the reference genome is available. Besides reducing the cost for organisms currently under intensive study (e.g. human, mouse), cuRRBS opens the door to the cost-effective study of DNA methylation in species with large genomes or where DNA methylation in non-CpG contexts is common, such as plants (58), which currently lack an MspI-based RRBS protocol, owing to the enzyme's CHG methylation sensitivity (59).

We decided to test the ability of cuRRBS to enrich for genomic sites that have important functional roles in different systems. Some of the systems that we tested *in silico* include genomic regions whose methylation status is important during cellular reprogramming (36), an epigenetic clock (37), transcription factor binding sites that are affected by DNA methylation (39,41), imprinted loci (35), CpGs found in the exon-intron boundaries and CHG sites that are differentially methylated between different arabidopsis accessions (38) (Supplementary Figure S4). For these *in silico* systems we chose to run the software with the *threshold* set to 25% of the maximum *Score*.

In all cases, cuRRBS is able to dramatically reduce the cost associated with the sequencing by several orders of magnitude compared to WGBS, which is assessed using the *Cost Reduction Factor* (*CRF*) (Supplementary Figure S5). In addition, for cases where a comparison to MspI-based RRBS could be made, cuRRBS is able to improve the *CRF*, again, by orders of magnitude. As an example, for the placental-specific imprints, the sequencing costs are reduced by approximately 400-fold when compared to WGBS and by 12.5-fold when compared to the traditional MspI-based RRBS.

Furthermore, we have also observed that many of the top hits reported by cuRRBS are digestions of two restriction enzymes (Supplementary Figure S4), highlighting the combinatorial power of restriction enzymes to produce optimal reduced representations of the genome (28). Excitingly, we are able to show that using cuRRBS it is possible to assay a far larger number of target sites, in a far simpler experimental design than would normally be achieved using amplicon-based bisulfite sequencing.

### Experimental validation of cuRRBS

To assess in an unbiased manner how well predictions from cuRRBS perform in an experimental setting, we employed two independent non-canonical RRBS datasets: one generated from a single enzyme (XmaI) and the other from a combination of two restriction enzymes (MspI and Taq^α^I) (30,31). By evaluating the predictive power of cuRRBS in these two datasets, we were able to observe cuRRBS’ performance in both single and double enzyme contexts and across different genomes.

**Figure 3.**
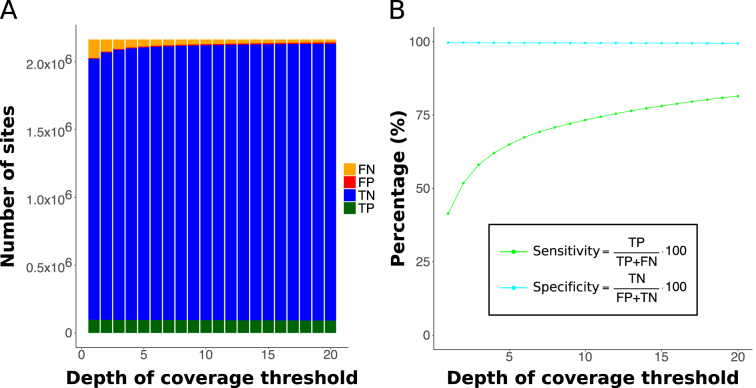
Experimental validation of cuRRBS. (**A**) Barplots showing the number of true positives (TP, in green), true negatives (TN, in blue), false positives (FP, in red) and false negatives (FN, in orange) when comparing cuRRBS theoretical prediction with the actual XmaI-RRBS experimental data (30). The number of sites in each category is calculated for different thresholds in the depth of coverage (number of reads covering a CpG site as reported by Bismark). cuRRBS prediction for the CpG sites in human CpG islands was obtained enforcing a theoretical size range of 90–185 bp and running the software for XmaI with all the default parameters (with a *read length* of 200 bp). Legend is displayed on the right hand side. (**B**) Plot showing values of cuRRBS sensitivity (in light green) and specificity (in cyan) as a function of the depth of coverage threshold employed to filter the experimental data (30). The number of true positives (TP), true negatives (TN), false positives (FP) and false negatives (FN) are the same as in A. Legend is displayed below the plot curves.

To test the accuracy of cuRRBS predictions in the context of a single enzyme digestion, we utilised the non-canonical RRBS dataset generated from human DNA using the restriction enzyme XmaI (30). This dataset was previously used to show that XmaI could enrich for CpG islands (CGIs), while reducing the overall sequencing cost relative to MspI, making the protocol more cost-effective. To validate cuRRBS using this system, we therefore chose to enrich for all CpG sites that overlapped with a CGI (CGI-CpGs) in the human genome using a predetermined theoretical size range equivalent to the ‘reproducible library fragment lengths’ reported in (30) (i.e. 90–185 bp). cuRRBS predicted with high accuracy the CpG sites that were observed in the experimental XmaI-RRBS dataset (Figure 3A). In particular, only a small proportion of the total number of CGI-CpGs should be theoretically sequenced (102 253 out of 2 164 614), and this was indeed the case (Figure 3A). Furthermore, upon filtering out sites with low depth of coverage, which commonly represent noise in RRBS datasets, the sensitivity increased up to ∼80%. Importantly, the specificity remained constant at ∼100% independent of the threshold set for depth of coverage (Figure 3B). Thus, cuRRBS produces a prediction that is relatively conservative, as highlighted by the low numbers of false positives (Figure 3A), at the expense of a small decrease in sensitivity.

Interestingly, the original theoretical size range that the study was aiming for (110–200 bp) was slightly different to the one achieved in the actual experiments (90–185 bp) (30). We ran cuRRBS using the original size range target and obtained slightly worse results for the sensitivity but not the specificity of the prediction (Supplementary Figure S6A and B). This demonstrates that the correct execution of the size selection step during the experimental protocol is key for obtaining the sites predicted by cuRRBS and highlights the importance of the *robustness* variable as part of the cuRRBS output in order to judge the consequences of these experimental errors.

To test the accuracy of cuRRBS predictions in the context of a double enzyme digestion, we utilised the non-canonical RRBS dataset generated from mouse DNA using the restriction enzymes MspI and Taq^α^I (31). To compare the accuracy of cuRRBS prediction in this double enzyme system to that of the XmaI-RRBS system, we again ran cuRRBS for CGI-CpGs, this time in the mouse genome with a theoretical size range of 80–160 bp (31). cuRRBS predicted with high accuracy the CpG sites that were observed in this double enzyme experiment (Supplementary Figure S6C). In addition, the results for sensitivity and specificity were very similar to the ones reported for the XmaI-RRBS dataset (Supplementary Figure S6D). Therefore, we conclude that cuRRBS produces robust predictions for the sites of interest that will be sequenced in RRBS protocols both for single and double enzyme combinations independent of the genome under study.

Lastly, the number of fragments that were theoretically recoverable in each of our experimental systems ranged from *NF* = 12 780 (for XmaI) to *NF* = 331 058 (for MspI and Taq^α^I). This represents approximately a 30-fold difference in the number of recoverable fragments and demonstrates that cuRRBS predictions, even for low *NF* values, are experimentally feasible. Importantly, in the nine theoretical examples that we report (Supplementary Figure S4), the number of fragments required by each cuRRBS protocol ranges from 107 248 to 974 050. Thus, the number of fragments required to achieve the stated *CRF* comfortably exceeds the minimum experimentally validated *NF* value (>8-fold).

## CONCLUSIONS AND FUTURE DIRECTIONS

cuRRBS provides a new framework that allows the user to optimise RRBS for the biological system of interest by using novel combinations of restriction enzymes. Therefore, cuRRBS makes the study of DNA methylation more affordable across all species for which genomic sequences are available. Furthermore, it can open the door to the design of future studies in a clinical context (10), which require cost-effective and robust protocols.

Currently, cuRRBS only considers combinations of up to two restriction enzymes. However, in the future, it would be possible to adapt the software to explore combinations that contain higher numbers of enzymes, which could theoretically allow targeting the sites of interest even more efficiently (28). Moreover, there are several methods that are able to impute DNA methylation levels in sites that are not covered experimentally (46,60). These methods could expand the set of sites of interest that are finally measured by making use of the additional DNA methylation information that is retrieved in a cuRRBS experiment.

Finally, the potential of restriction enzymes to target different genomic coordinates is not limited to DNA methylation. As such, it would be conceivable for cuRRBS to be adapted to enrich for SNPs of interest (61,62) or to optimise chromosome conformation capture techniques (63,64). By reducing the cost associated with sequencing, we believe that cuRRBS will help to democratise high-throughput genomic studies.

## DATA AVAILABILITY

cuRRBS and its documentation are freely distributed under GNU General Public License v3.0 and can be accessed via GitHub (51). The public RRBS data used in this manuscript can be found under GEO accession numbers GSE74126 (XmaI-RRBS) and GSE80961 (MspI&Taq^α^I-RRBS) respectively.

## Supplementary Material

Supplementary Data
