# Supplementary material for: cuRRBS: simple and robust evaluation of enzyme combinations for reduced representation approaches
Source: Nucleic Acids Res. 2017 Sep 19;45(20):11559–69. doi: 10.1093/nar/gkx814 (PMC5714207; doi:10.1093/nar/gkx814)
Supplement: Supplementary Data [file gkx814_supp.zip › nar-02012-n-2017-File005.pdf]

Supplementary Figures and Tables

cuRRBS: simple and robust evaluation of enzyme combinations for reduced representation approaches

Daniel E. Martin-Herranz[1,*], Antonio J. M. Ribeiro[1], Felix Krueger[2], Janet M. Thornton[1,†], Wolf Reik[3,4,5,†], Thomas M. Stubbs[3,*,†]

[1] European Molecular Biology Laboratory, European Bioinformatics Institute, Wellcome Genome Campus, Hinxton CB10 1SD, UK.
[2] Bioinformatics Group, The Babraham Institute, Cambridge CB22 3AT, UK.
[3] Epigenetics Programme, The Babraham Institute, Cambridge CB22 3AT, UK.
[4] Centre for Trophoblast Research, University of Cambridge, Cambridge CB2 3EG, UK.
[5] Wellcome Trust Sanger Institute, Hinxton CB10 1SA, UK.

[*]Equal contribution

[†]Correspondence:
thornton@ebi.ac.uk
wolf.reik@babraham.ac.uk
Thomas.stubbs@babraham.ac.uk

Author email addresses:
dem44@ebi.ac.uk
ribeiro@ebi.ac.uk
felix.krueger@babraham.ac.uk

Supplementary Figure 1

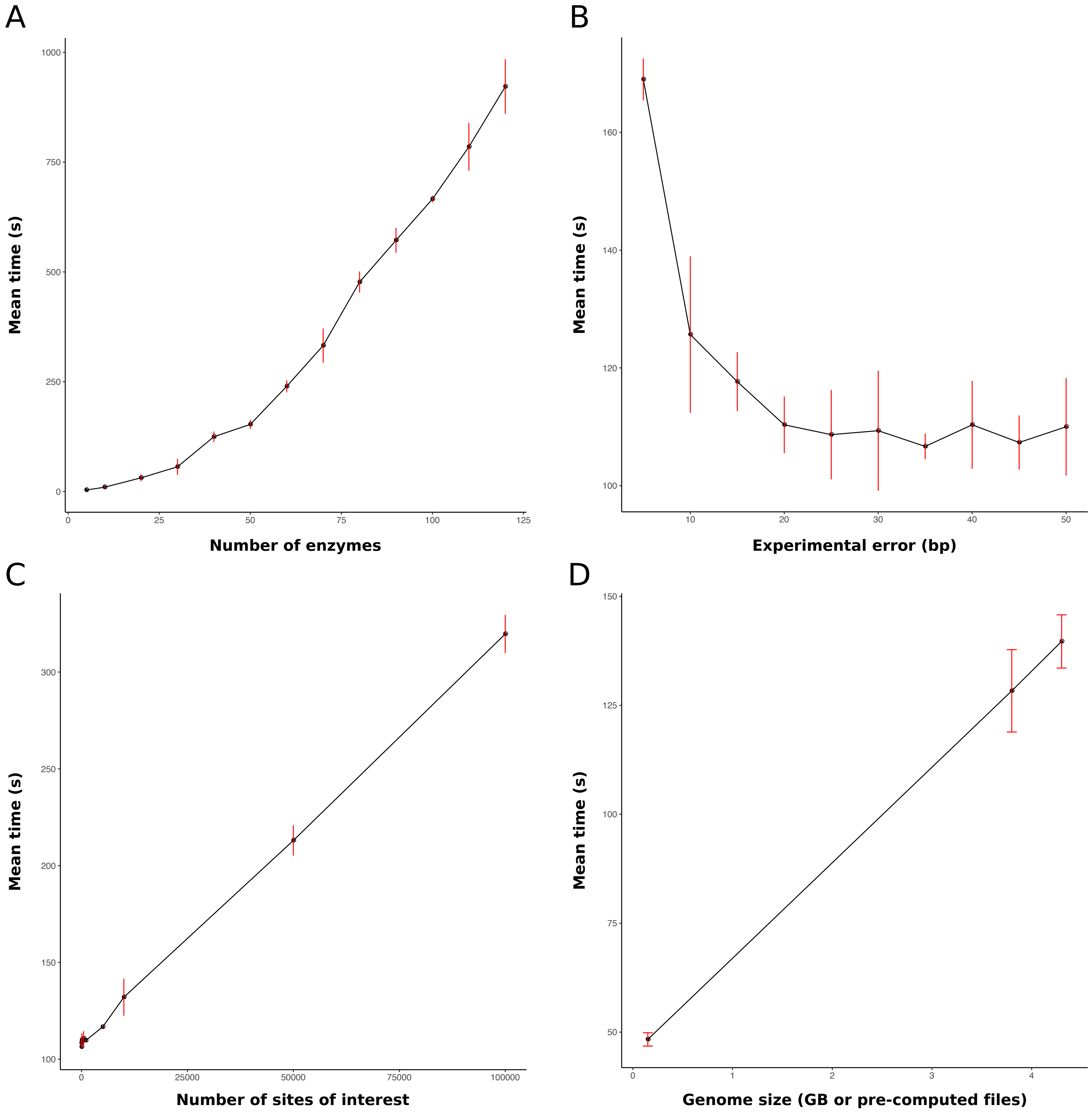

Supplementary Figure 1: cuRRBS computational efficiency.

A. Plot showing the dependency between the number of enzymes checked and the computational (real) time required by the software (mean between 3 independent runs). cuRRBS was run for the human epigenetic clock system (37) with a *read length* of 75 bp, a *Score threshold* of 25 % and an *experimental error* of 10 bp. A laptop with an Intel® Core™ i7-6600U CPU was used, which allowed cuRRBS to employ 4 parallel threads. The red error bars display the mean ± s.d. for the 3 independent runs.

B. Plot showing the dependency between the *experimental error* (which determines how many size ranges are sampled) and the computational (real) time required by the software (mean between 3 independent runs). cuRRBS was run for the human epigenetic clock system (37) with a *read length* of 75 bp, a *Score threshold* of 25 % and a list with 40 enzymes. A laptop with an Intel® Core™ i7-6600U CPU was used, which allowed cuRRBS to employ 4 parallel threads. The red error bars display the mean ± s.d. for the 3 independent runs.

C. Plot showing the dependency between the number of sites of interest and the computational (real) time required by the software (mean between 3 independent runs). cuRRBS was run with a *read length* of 75 bp, a *Score threshold* of 25 %, an *experimental error* of 10 bp and a list with 40 enzymes. A laptop with an Intel® Core™ i7-6600U CPU was used, which allowed cuRRBS to employ 4 parallel threads. The red error bars display the mean ± s.d. for the 3 independent runs.

D. Plot showing the dependency between genome size (measured as the size in GB of all the pre-computed files) and the computational (real) time required by the software (mean between 3 independent runs). cuRRBS was run with a *read length* of 75 bp, a *Score threshold* of 25 %, an *experimental error* of 10 bp and a list with 40 enzymes. A laptop with an Intel® Core™ i7-6600U CPU was used, which allowed cuRRBS to employ 4 parallel threads. The red error bars display the mean ± s.d. for the 3 independent runs.

Supplementary Figure 2

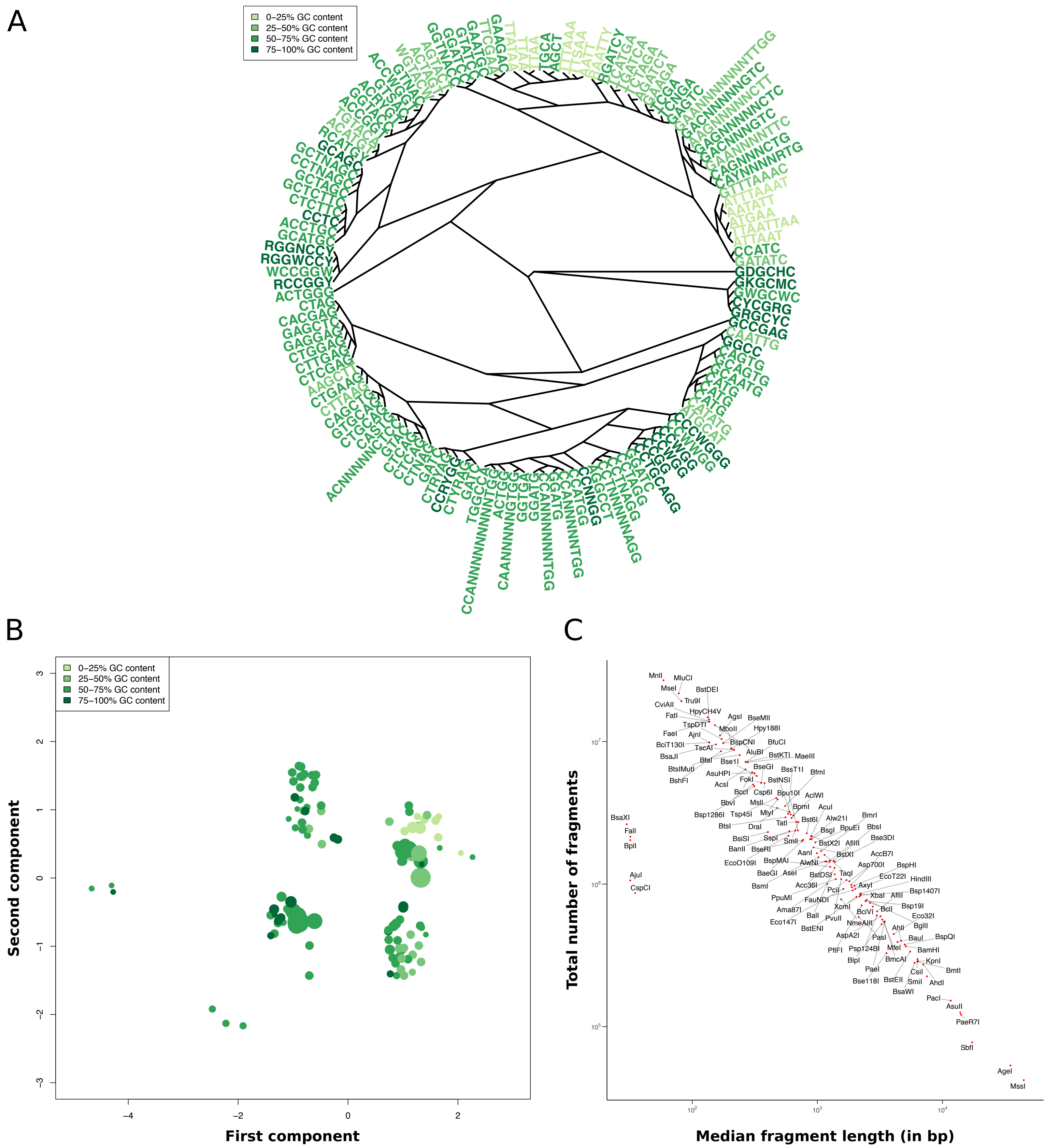

Supplementary Figure 2

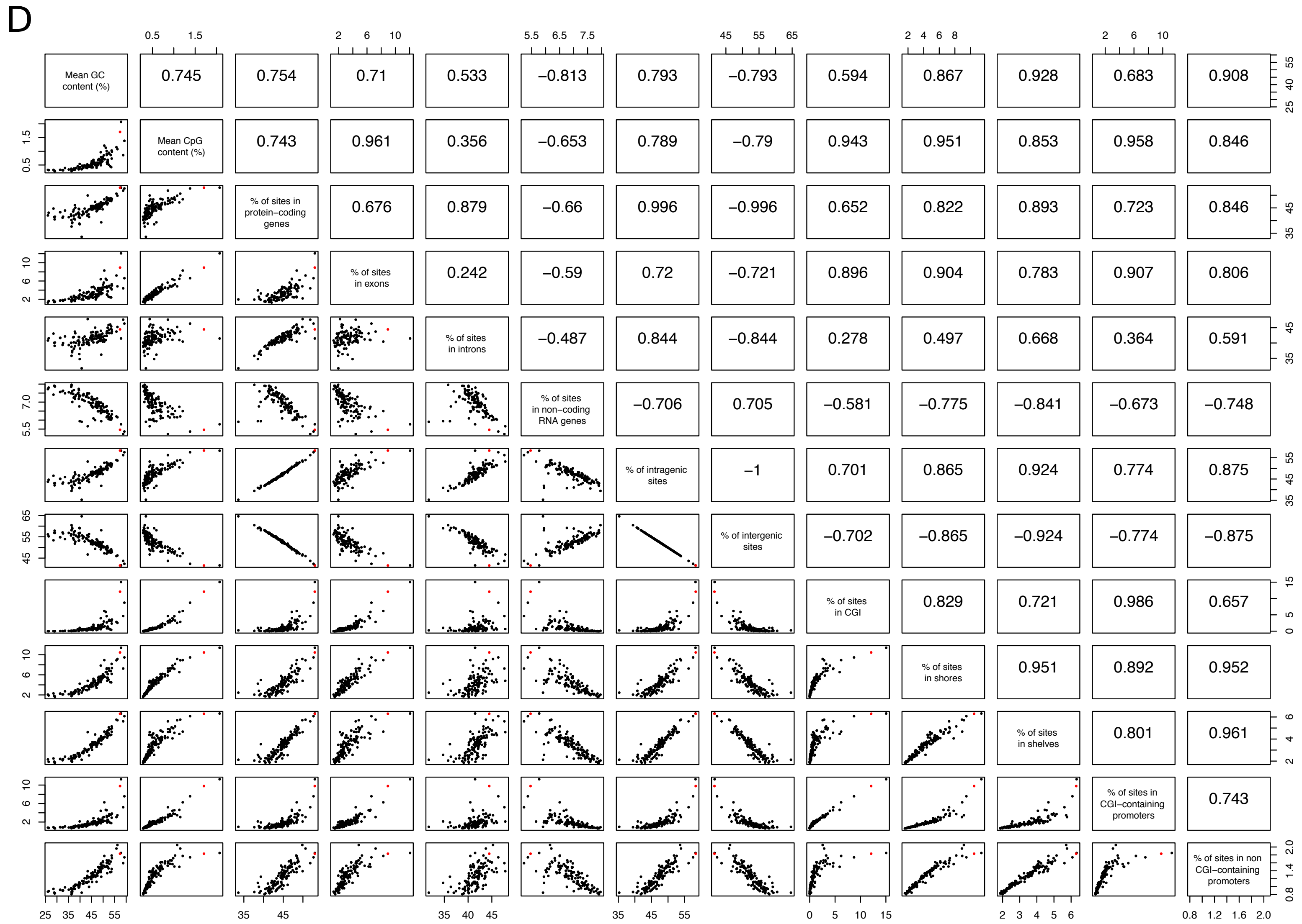

Supplementary Figure 2

E

First author(s)	Title	Date	Single enzymes checked	Double enzymes checked	Size ranges interrogated	Genomic regions targeted	Organism(s)	Read lengths tested	For sequencing	Code available
Cedar H	Direct detection of methylated cytosine in DNA by use of the restriction enzyme MspI	1979	YES	NO	NA	NA	*Neurospora crassa*, herpes virus, fly, bovine	NA	N	N
Yu L	A NotI–EcoRV promoter library for studies of genetic and epigenetic alterations in mouse models of human malignancies	2004	YES	YES	NA	CpG islands, protein-coding genes	Human (hg16), mouse (mm4)	NA	Y	N
Wang J and Xia Y	Double restriction-enzyme digestion improves the coverage and accuracy of genome-wide CpG methylation profiling by reduced representation bisulfite sequencing	2013	YES	YES	2	Increase CpG coverage genome-wide	Human (hg18), mouse(mm9)	50 bp PE, 90 bp PE	Y	N
Bystrykh L	A combinatorial approach to the restriction of a mouse genome	2013	YES	YES	NA	NA	Mouse (mm10)	NA	N	N
Martinez-Arguelles DB	In silico analysis identifies novel restriction enzyme combinations that expand reduced representation bisulfite sequencing CpG coverage	2014	YES	YES	1	Increase CpG coverage genome-wide	Human (hg38), mouse (mm10), rat (NCBI build 4.2)	50 bp PE	Y	N
Lee YK and Jin S	Improved reduced representation bisulfite sequencing for epigenomic profiling of clinical samples	2014	YES	YES	1	Increase CpG coverage genome-wide	Human (hg19)	36 bp PE	Y	N
Kirschner SA	Focussing reduced representation CpG sequencing through judicious restriction enzyme choice	2016	YES	YES	2	Increase CpG coverage genome-wide	Mouse (mm10)	NA	Y	N
Tanas AS	Rapid and affordable genome-wide bisulfite DNA sequencing by XmaI-reduced representation bisulfite sequencing	2017	YES	NO	1	CpG islands	Human (hg19)	NA	Y	N
Stubbs TM and Martin-Herranz DE	cuRRBS	2017	YES	YES	Defined by the user	Defined by the user	Defined by the user	Defined by the user	Y	Y

Supplementary Figure 2: complementary to Figure 1.

A. Phylogenetic analysis of the motifs that are recognised by the different commercially-available restriction enzymes which are insensitive to CpG methylation. Each sequence represents a different isoschizomer family considered in this study. A neighbour-joining method was used to construct the tree. Motifs with different GC content are shown with different colours.

B. Principal components analysis (PCA) performed on the matrix of pairwise distances from the aligned motifs (same as in Supplementary Figure 2A). Each circle represents a different motif. The coordinates of the different motifs on the first two principal components are plotted on the X- and Y-axes. Motifs with different GC content are shown with different colours and the motif length is represented by the diameter of the circle.

C. Scatterplot which summarises the fragment length distributions for the same isoschizomer families portrayed in Figure 1A. The red dots represent the actual values of median fragment length and total number of fragments for each family. The black lines assign each name label to the correspondent red point for visualization purposes.

D. Matrix of scatterplots showing the percentages of cleavage sites from different restriction enzymes that overlap with several genomic features (listed on the diagonal) in the human genome (hg38). The red dot in each scatterplot represents the values for MspI. The numbers above the diagonal are the Pearson correlation coefficients between all the possible pairs of genomic features.

E. Table showing the comparison of different studies that have attempted to use restriction enzymes to target different regions in the genome.

Supplementary Figure 3

A

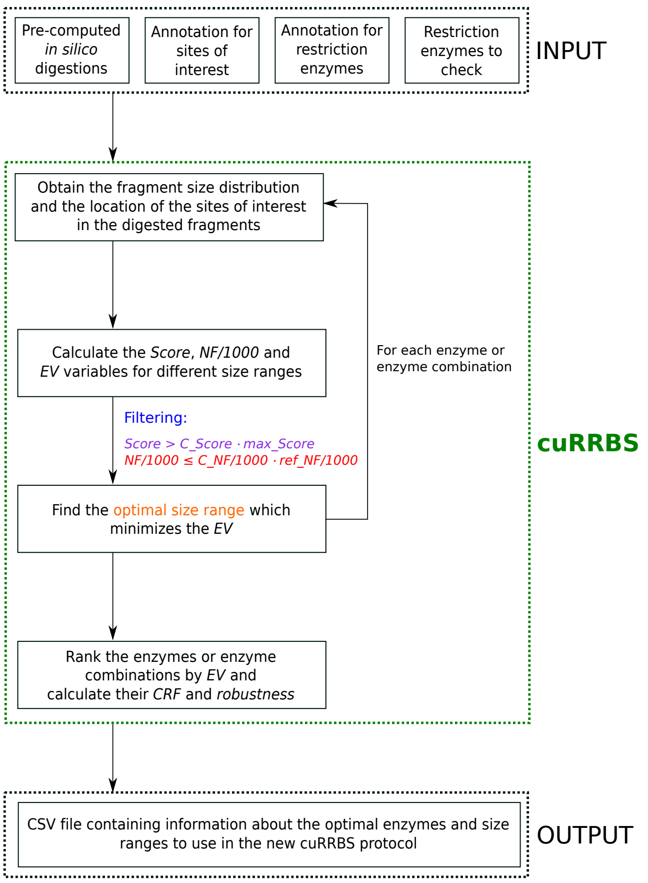

B

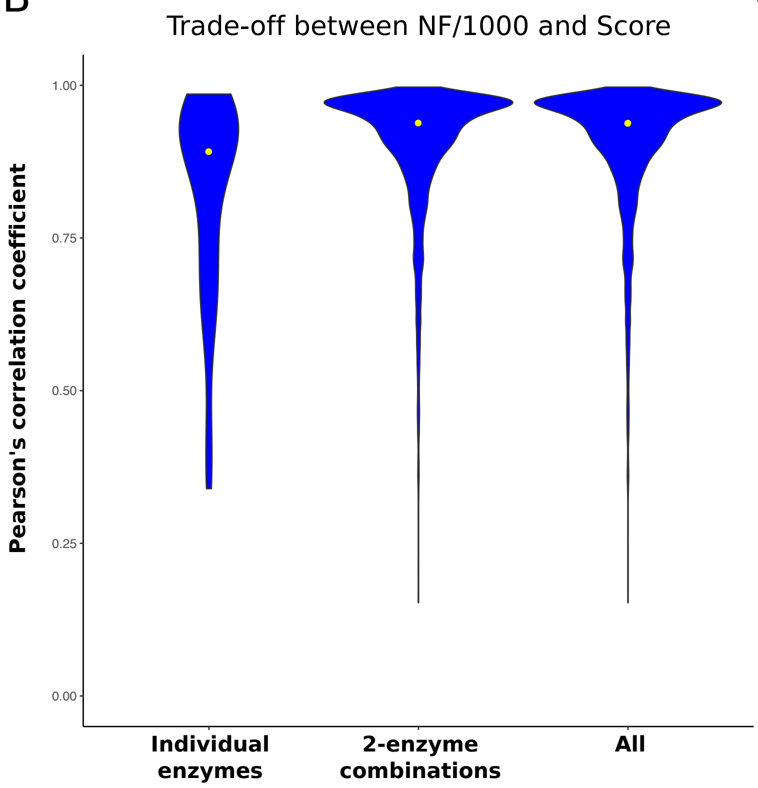

C

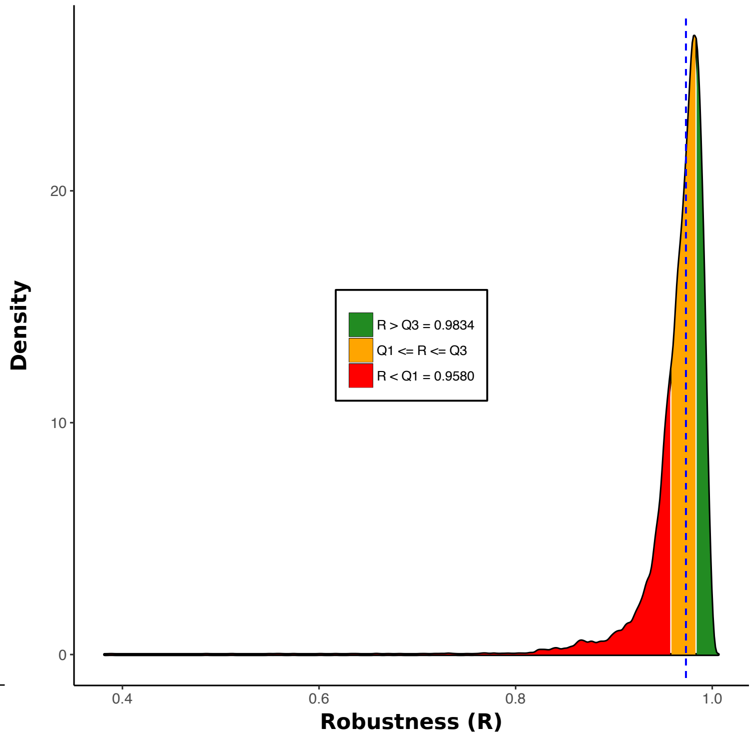

Supplementary Figure 3: complementary to Figure 2.

A. Detailed flowchart showing the input, main steps in cuRRBS and the output of the software.

B. Violin plots showing the distribution of Pearson correlation coefficients between the number of fragments (*NF*) and the *Score* for all the different enzymes tested with cuRRBS (single-enzyme, double-enzyme, all). In this example we used the human epigenetic clock system (37), checking all the *size ranges* between 20 and 1000 bp, with an *experimental error* of 10 bp and a *read length* of 75 bp. Each yellow point represents the median for the Pearson correlation coefficients under consideration.

C. Density plot showing the distribution of the *robustness* (*R*) values when assuming an *experimental error* (δ) of 20 bp. cuRRBS was run for all the biological systems under study (Supplementary Figure 4) (35-41) with the same parameters as described in 'Running cuRRBS for different *in silico* systems' (all the hits that satisfied the *thresholds* were reported in this case). The dashed blue line represents the median (0.9734). The different colours provide a way to judge the *robustness* values: bad (in red, $R < Q_1 = 0.9580$), medium (in orange, $Q_1 \leq R \leq Q_3 = 0.9834$) and good (in green, $R > Q_3$); where Q_1 and Q_3 represent the first and the third quartiles respectively.

Supplementary Figure 4

**Species**	**System (as shown in Figure 3)**	**PMID where applicable**	**Additional information about the system**	**Total number of sites targeted**	**Optimal restriction enzyme combination**	**Optimal theoretical size range (in bp)**	**% max Score**	**NF/1000**	**Enrichment Value (EV)**	**Cost Reduction Factor (CRF)**	**Robustness (R)**
Homo sapiens	Exon-intron boundaries		DNA methylation has been shown to affect alternative splicing. Therefore, we focused on targeting CpGs close to canonical splicing sites.	26211	(BsiSI OR MspI) AND (SbfI OR SdaI OR Sse8387I)	80_500	25.4	772.23	2.064468112	53.32	0.94704403
Homo sapiens	Epigenetic clock	24138928	The Horvath epigenetic clock is the best predictor of biological age available in humans. We have attempted to target the 353 CpG sites that are used in the model in order to reduce the cost associated with the assay.	353	(BsiSI OR MspI) AND (BspQI OR LguI OR SapI)	60_160	27.57	442.456	3.657719163	93.06	0.91305072
Homo sapiens	Imprinted loci	26769960	Genomic imprinting is an epigenetic phenomenon that results in gene expression occuring in a parent-of-origin fashion. We have attempted to target Cs in CpG context that are found within the canonical human imprints.	2810	(BmeT110I OR BsoBI) AND (BsaWI)	60_540	25.12	336.88	2.678670527	122.23	0.98085689
Homo sapiens	Placental imprinted loci	26769960	Genomic imprinting is an epigenetic phenomenon that results in gene expression occuring in a parent-of-origin fashion. However, until recently many extraembryonic imprints were still unknown. We have targetted Cs in CpG context that are found within these novel human placental imprints.	7591	(BsaWI) AND (BssAI)	60_540	26.41	107.248	1.728274825	383.94	0.93382453
Homo sapiens	CTCF sites	26257180	CTCF is an important architectural protein that helps to organise chromatin domains. Since its binding has been shown to be dependent on DNA methylation in some of its recognition sequences, we have targeted the CpG sites within these regions of the genome.	2000	(BmeT110I OR BsoBI) AND (BssAI)	40_360	25.5	314.079	2.789468723	131.1	0.88798165
Mus musculus	iPSCs demethylated	28147265	iPSC reprogramming in mouse is characterised by global changes in DNA methylation. Sites that tend to undergo demethylation faster than the genome average tend to be within ESC-Super Enhancers. We targetted the Cs in CpG context in these regions, as they are interesting for the reprogramming field.	1449	(BmeT110I OR BsoBI) AND (BsiSI OR MspI)	80_980	25.19	974.05	3.426288386	37.31	0.96792238
Mus musculus	iPSCs maintained	28147265	iPSC reprogramming in mouse is characterised by global changes in DNA methylation. Sites that tend to be resistant to the genome-wide demethylation tend to be within Intercisernal A-particle containing regions. We targetted the Cs in CpG context in these regions, as they are interesting for the reprogramming field.	3896	(BmeT110I OR BsoBI) AND (BsiSI OR MspI)	80_560	25.85	690.088	2.835875005	52.66	0.94227711
Mus musculus	NRF1 sites	26675734	NRF1 is a transcription factor whose binding to the DNA is dependent on the methylation status of its recognition sequences. We have tried to enrich for those CpG sites that overlap with *in vivo* NRF1 binding sites.	17018	(BmeT110I OR BsoBI) AND (PaeI OR SphI)	20_760	25.04	445.36	2.019097763	81.6	0.99634045
Arabidopsis thaliana	CHG sites	27419873	Non-CpG methylation is an important epigenetic modification in plants. In this study a huge number of regions containing non-CpG methylation were found to vary between different Arabidopsis accessions in the 1001 Epigenomes Project. We targetted Cs in non-CpG context within these non-CpG DMRs.	21801	(AanI OR PsiI) AND (Csp6I OR CviQI)	100_520	25.05	165.313	1.480955311	9.65	0.94999336

Supplementary Figure 4: Running cuRRBS in different biological systems.

Table showing the information regarding the different biological systems (35-41) for which cuRRBS was run *in silico*. Some variables from the top hits in cuRRBS output are also reported.

Supplementary Figure 5

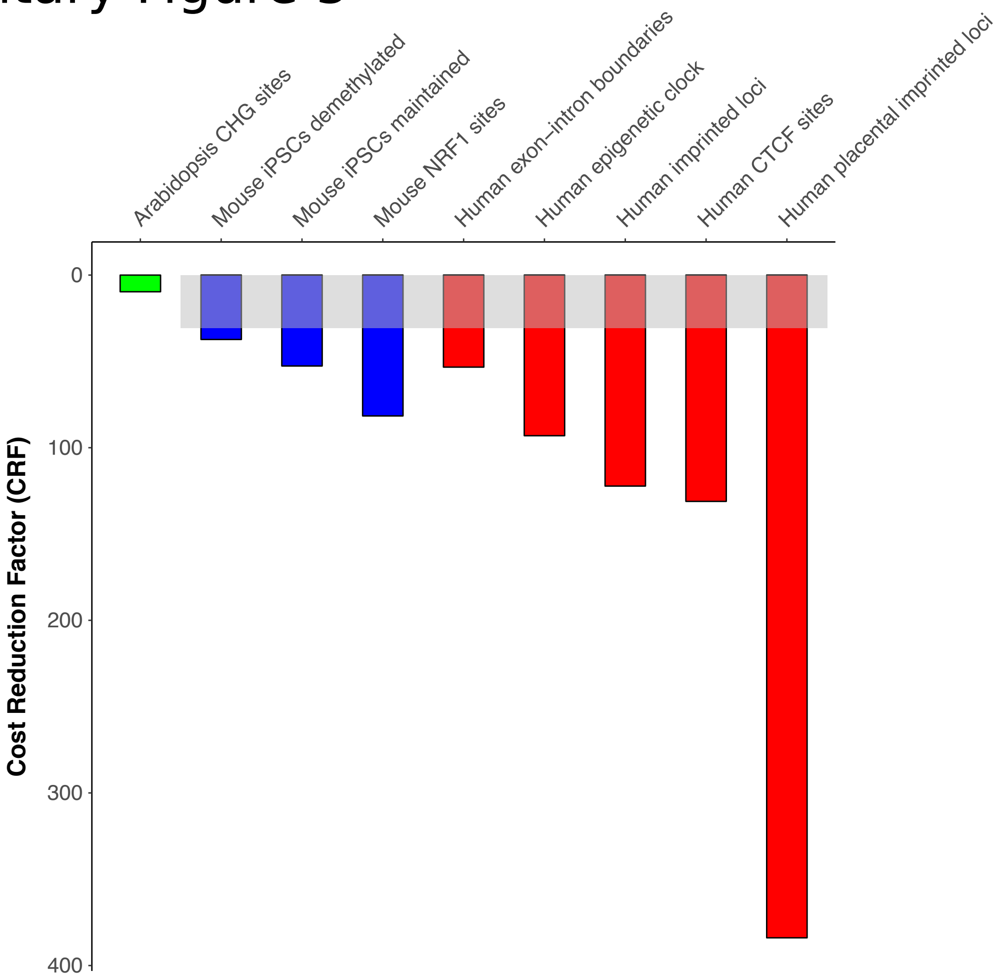

Supplementary Figure 5: Running cuRRBS in different biological systems.

Barplot showing the values for the *Cost Reduction Factor* (*CRF*) in the different biological systems that were tested (see Supplementary Figure 4) (35-41). The colours in the bars represent the different species interrogated (green: *Arabidopsis thaliana*, blue: *Mus musculus*, red: *Homo sapiens*). The *CRF* for the traditional RRBS protocol (MspI in the human genome, using a bead size selection step of 20-800 bp*, CRF* = 30.65) is displayed as a grey area, which is not compared with the *A. thaliana* system (since MspI is sensitive to CHG methylation).

Supplementary Figure 6

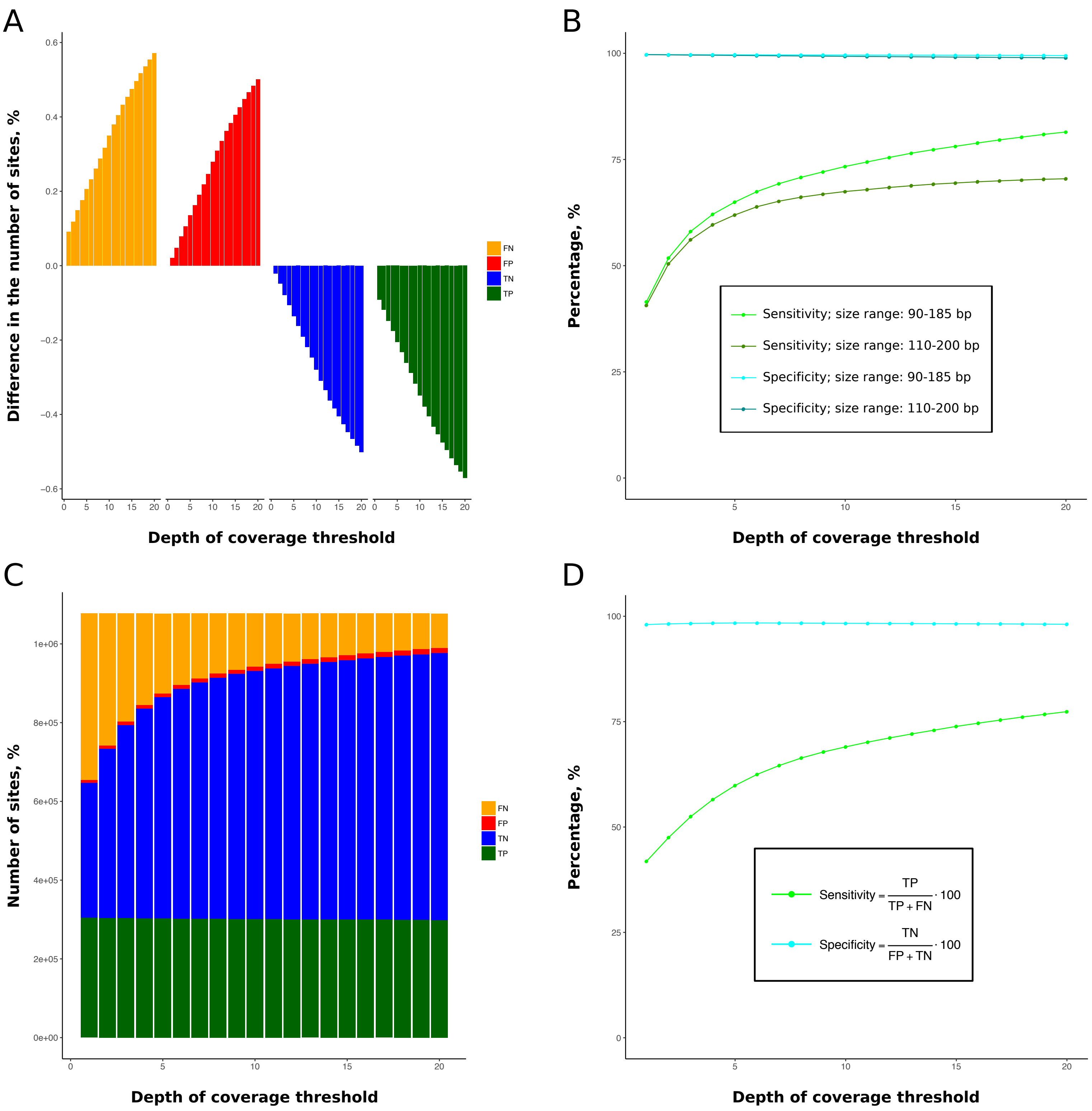

Supplementary Figure 6: complementary to Figure 3.

A. Barplots showing the difference in the number of true positives (TP, in green), true negatives (TN, in blue), false positives (FP, in red) and false negatives (FN, in yellow) derived from cuRRBS theoretical predictions for the XmaI-RRBS data (30) using two different size ranges: 110-200 bp (aimed size range) and 90-185 bp (real size range). The difference observed between the two size ranges (aimed - real) is expressed as the percentage of the total number of sites considered (i.e. all CGI-CpGs). The number of sites in each category is calculated for different thresholds in the depth of coverage (number of reads covering a CpG site as reported by Bismark). cuRRBS was run for XmaI with all the default parameters (with a *read length* of 200 bp). Legend is displayed on the right hand side.

B. Plot showing values of cuRRBS sensitivity and specificity as a function of the depth of coverage threshold employed to filter the experimental data (30). The two size ranges considered in Supplementary Figure 6A (aimed: 110-200 bp; real: 90-185 bp) are used for the calculations. Legend is displayed below the plot curves.

C. Barplots showing the number of true positives (TP, in green), true negatives (TN, in blue), false positives (FP, in red) and false negatives (FN, in orange) when comparing cuRRBS theoretical prediction with the actual MspI&Taq$^{\alpha}$I-RRBS experimental data (31). The number of sites in each category is calculated for different thresholds in the depth of coverage (number of reads covering a CpG site as reported by Bismark). cuRRBS prediction for the CpG sites in mouse CpG islands was obtained enforcing a theoretical size range of 80-160 bp and running the software for MspI&Taq$^{\alpha}$I with all the default parameters (with a *read length* of 75 bp). Legend is displayed on the right hand side.

D. Plot showing values of cuRRBS sensitivity (in light green) and specificity (in cyan) as a function of the depth of coverage threshold employed to filter the experimental data (31). The number of true positives (TP), true negatives (TN), false positives (FP) and false negatives (FN) are the same as in Supplementary Figure 6C. Legend is displayed below the plot curves.

Supplementary Tables

Supplementary Table 1

A comma-separated values (CSV) file that contains the annotation for the CpG sites found in the human exon-intron boundaries. The different columns represent site ID, chromosome, genomic coordinate and weight for each one of the sites of interest (rows). Equal weights ($w_i = 1$) were assigned to all the sites.

Supplementary Table 2

A comma-separated values (CSV) file that contains the annotation for the CpG sites found in the human epigenetic clock (37). The different columns represent site ID, chromosome, genomic coordinate and weight for each one of the sites of interest (rows). The sites were assigned weights equivalent to the absolute values of the weights in the linear model.

Supplementary Table 3

A comma-separated values (CSV) file that contains the annotation for the CpG sites found in the human canonical imprinted loci (35). The different columns represent site ID, chromosome, genomic coordinate and weight for each one of the sites of interest (rows). Equal weights ($w_i = 1$) were assigned to all the sites.

Supplementary Table 4

A comma-separated values (CSV) file that contains the annotation for the CpG sites found in the human placental-specific imprinted loci (35). The different columns represent site ID, chromosome, genomic coordinate and weight for each one of the sites of interest (rows). Equal weights ($w_i = 1$) were assigned to all the sites.

Supplementary Table 5

A comma-separated values (CSV) file that contains the annotation for the CpG sites that overlap with *in vivo* CTCF binding sites in human (39). The different columns represent site ID, chromosome, genomic coordinate and weight for each one of the sites of interest (rows). Equal weights ($w_i = 1$) were assigned to all the sites.

Supplementary Table 6

A comma-separated values (CSV) file that contains the annotation for the CpG sites found to actively demethylate during iPSC reprogramming in mouse (36). The different columns represent site ID, chromosome, genomic coordinate and weight for each one of the sites of interest (rows). Equal weights ($w_i = 1$) were assigned to all the sites.

Supplementary Table 7

A comma-separated values (CSV) file that contains the annotation for the CpG sites found to resist demethylation during iPSC reprogramming in mouse (36). The different columns represent site ID, chromosome, genomic coordinate and weight for each one of the sites of interest (rows). Equal weights ($w_i = 1$) were assigned to all the sites.

Supplementary Table 8

A comma-separated values (CSV) file that contains the annotation for the CpG sites that overlap with *in vivo* NRF1 binding sites in mouse (41). The different columns represent site ID, chromosome, genomic coordinate and weight for each one of the sites of interest (rows). Equal weights ($w_i = 1$) were assigned to all the sites.

Supplementary Table 9

A comma-separated values (CSV) file that contains the annotation for the CHG sites found to be differentially methylated between different accessions in *Arabidopsis thaliana* (38). The different columns represent site ID, chromosome, genomic coordinate and weight for each one of the sites of interest (rows). Equal weights (w_i = 1) were assigned to all the sites.
